# Supplementary material for: Flow characterization and structural alterations in Ahmed glaucoma FP7 tubes after in-vitro aging in silicone oil
Source: PLoS One. 2024 Nov 5;19(11):e0310564. doi: 10.1371/journal.pone.0310564 (PMC11537406; doi:10.1371/journal.pone.0310564)
Supplement: Attachment — Submitted filename: PONE-D-24-14929.docx [file pone.0310564.s002.docx]

**PONE-D-24-14929**

The manuscript presents findings from an experimental study investigating the impact of prolonged exposure to silicone oil (SO) on silicone-based Ahmed Glaucoma Valve (AGV) tubes. The study suggests that SO exposure may enhance flow due to lumen diameter expansion, challenging previous assumptions about its negative impact. However, several critical issues in organization, clarity, methodology, and presentation must be addressed before publication.

**Specific Comments:**

1. **Title and Abstract:**
   - The study's title must accurately represent the research's full scope. It only focuses on flow characterization and does not represent the physical changes involved.
   - The abstract needs more substance. It fails to emphasize the significance of the research and presents the key findings in an unclear manner. It should be revised to clearly communicate the study's purpose, methods, key findings, and implications.
2. **Introduction:**
   - The introduction needs more organization and depth. It should provide a more profound background on AGVs, their failure mechanisms in the presence of silicone oil, and the gaps in the current literature that the study aims to address. This would better orient readers and justify the study's importance.
3. **Methodology:**
   - The rationale for the 90.5-day aging period needs more explanation and seems arbitrary. Consider providing a rationale based on previous literature or preliminary studies.
   - Including controls with the different viscosities of silicone oil at 37°C could elucidate the effects observed and provide a more comprehensive analysis.
   - The lack of flow control valves limits the study's conclusions regarding the impact of silicone oil on AGV flow rates. Consider revising the experimental setup to address this limitation or explain why only the tubes are being subject to study.
   - A more suitable model for this experimental setup would involve using different types of GDD, such as the Baerveldt implant, which lacks a valvular design.
   - Inconsistent readout terminology (e.g., inner lumen edge, outer lumen edge, outer tube area) should be clarified and consistently used throughout the manuscript and figures.
4. **Results:**
   - The results section is confusing and overly reliant on figures. It needs restructuring with clearer organization and precise statistical explanations to enhance readability and interpretation, other than just stating “percentage changes.”
5. **Discussion:**
   - The discussion compares findings with existing literature but needs to state how this study contributes to the field. Emphasizing the clinical implications of glaucoma treatment is essential.
   - The limitation regarding flow rate findings is identified but not thoroughly discussed. To strengthen the discussion section, this should be addressed.
6. **Conclusion:**
   - The conclusion must be revised, and the study's findings must be summarized or suggested for future research directions. It should be rewritten to encapsulate the study's contributions, implications, and areas for further investigation.
7. **Overall Quality:**
   - The manuscript suffers from grammatical errors, lack of coherence, and poor organization, undermining its clarity and impact. A thorough revision is needed to improve the logical flow from the introduction to the conclusion.
   - Figures effectively present results but suffer from inconsistent legend terminology, which needs to be corrected to prevent reader confusion.

**Recommendation:** The manuscript presents potentially valuable insights but requires significant revisions to meet the standards for publication. Addressing the abovementioned issues will strengthen the manuscript's clarity, coherence, and impact. Specifically, enhancing the abstract, introduction, methodology, results, discussion, and conclusion sections while ensuring consistency in terminology and clarity of presentation will significantly improve the manuscript's quality.

**Summary:** This manuscript investigates the effects of silicone oil on AGV tubes, suggesting potential benefits for flow enhancement. However, critical issues in organization, clarity, methodology, and presentation detract from its impact and suitability for publication in its current form. Significant revisions are necessary to address these concerns and improve the manuscript's quality and coherence.
