# Supplementary material for: Flow characterization and structural alterations in Ahmed glaucoma FP7 tubes after in-vitro aging in silicone oil
Source: PLoS One. 2024 Nov 5;19(11):e0310564. doi: 10.1371/journal.pone.0310564 (PMC11537406; doi:10.1371/journal.pone.0310564)
Supplement: Attachment — Submitted filename: response to reviewers_v2.docx [file pone.0310564.s003.docx]

**POINT-BY-POINT RESPONSE FORM**

Manuscript #: **PONE-D-24-14929**

Manuscript title: Flow characterization and structural alterations in Ahmed glaucoma FP7 tubes after in-vitro aging in silicone oil

| **Suggestion, Question,**  **or Comment from**  **Reviewer #2** | **Author’s Response** | **Change in the Manuscript** |
| --- | --- | --- |
| 1. The manuscript presents findings from an experimental study investigating the impact of prolonged exposure to silicone oil (SO) on silicone-based Ahmed Glaucoma Valve (AGV) tubes. The study suggests that SO exposure may enhance flow due to lumen diameter expansion, challenging previous assumptions about its negative impact. However, several critical issues in organization, clarity, methodology, and presentation must be addressed before publication.  **Title and Abstract:**  2. The study's title must accurately represent the research's full scope. It only focuses on flow characterization and does not represent the physical changes involved.  3. The abstract needs more substance. It fails to emphasize the significance of the research and presents the key findings in an unclear manner. It should be revised to clearly communicate the study's purpose, methods, key findings, and implications.  **Introduction:**  4. The introduction needs more organization and depth. It should provide a more profound background on AGVs, their failure mechanisms in the presence of silicone oil, and the gaps in the current literature that the study aims to address. This would better orient readers and justify the study's importance.  **Methodology:**  5. The rationale for the 90.5-day aging period needs more explanation and seems arbitrary. Consider providing a rationale based on previous literature or preliminary studies.  6. Including controls with the different viscosities of silicone oil at 37°C could elucidate the effects observed and provide a more comprehensive analysis.  7. The lack of flow control valves limits the study's conclusions regarding the impact of silicone oil on AGV flow rates. Consider revising the experimental setup to address this limitation or explain why only the tubes are being subject to study.  8. A more suitable model for this experimental setup would involve using different types of GDD, such as the Baerveldt implant, which lacks a valvular design.  9. Inconsistent readout terminology (e.g., inner lumen edge, outer lumen edge, outer tube area) should be clarified and consistently used throughout the manuscript and figures.  **Results:**  10. The results section is confusing and overly reliant on figures. It needs restructuring with clearer organization and precise statistical explanations to enhance readability and interpretation, other than just stating “percentage changes.”  **Discussion:**  11. The discussion compares findings with existing literature but needs to state how this study contributes to the field. Emphasizing the clinical implications of glaucoma treatment is essential.  12. The limitation regarding flow rate findings is identified but not thoroughly discussed. To strengthen the discussion section, this should be addressed.  13. The conclusion must be revised, and the study's findings must be summarized or suggested for future research directions. It should be rewritten to encapsulate the study's contributions, implications, and areas for further investigation.  **Overall Quality:**  14. The manuscript suffers from grammatical errors, lack of coherence, and poor organization, undermining its clarity and impact. A thorough revision is needed to improve the logical flow from the introduction to the conclusion.  15. Figures effectively present results but suffer from inconsistent legend terminology, which needs to be corrected to prevent reader confusion. | 1. Dear reviewer, thank you for your helpful comments and detailed insights. Below you will find our response to each of your comment.  2. We have replaced the title to reflect the entire scope of the study.  3. We have thoroughly reviewed and revised our abstract to enhance the clarity of each sub-section.  4. We have re-structured the introduction section to improve organization and included more background about AGV and their failure mechanism in eyes with SO instillation. We have explicitly stated the gap in current literature and how our study aims to fulfil that gap.  5. 90.5 day aging period was chosen based on the clinical practice patterns of retina specialists, most of whom use SO tamponade for a duration of 30-60 days. We have added this information in the methods section along with pertinent reference.  6. The purpose of including BSS controls at 37°C was to account for any stochastic effects resulting from putting tubes in an incubator for 13 days. Seeing at those tubes did not display any significant dimensional changes after aging, the authors did not deem it necessary to expend a limited resource on gaining additional data points that would not be clinically meaningful since 13 days of SO endotamponade is not a routine clinical practice.  7. Glaucoma drainage devices (GDD) tube shunts are usually inserted 1.5 – 2 mm into the anterior chamber or 3.5 – 4 mm for pars plana insertion. As such, we chose only tube sections for our analysis because they are most likely to encounter silicone oil and undergo structural changes by the interaction. Several studies confirm this by reporting silicone oil to be either adherent to the tube shunt or migrating inwards. The authors also arranged a meeting with the scientists at the New World Medical, Inc to perform flow tests using the entire AGV by utilizing their proprietary instruments but were ultimately denied access. We discuss this an important work for future studies.  8. The key purpose of our study was to ascertain the impact of silicone oil on silicone-based tubes of AGV. We agree with the reviewer that having various types of GDDs would add further generalize the results. However, this aspect of the experiment was limited by the ability of various companies to donate GDDs for our study. It may be important to note that most GDDs share a common design of silicone-based tube shunt, although the rest of the design may vary (1). Ultimately, inter-device comparability of flowrates is limited by various other factors such as surgical technique, goodness of design of the GDD, and patient specific factors. Therefore, the authors believe that a future study utilizing an in vitro eye model would be better suited to accomplish that aim. We are including reviewer’s comment as a limitation in our discussion.  Reference:  1. Agrawal P, Bhardwaj P. Glaucoma drainage implants. Int J Ophthalmol. 2020;13(8):1318-1328.  9. We have revised this as “inner lumen area” and “outer lumen area” throughout the manuscript and figures.  10. The results have been restructured to include the effect sizes of the changes in physical dimensions and flow rates paired with their statistical significance.  11. We have added a discussion of the clinical implications of our results.  12. We have discussed this limitation further.  13. We have revised the conclusion accordingly.  14. We have revised each section to improve the coherence and logical flow and fixed any grammatical errors.  15. Please see our response to comment #9. | 1. None  2. Lines 2-3 of the submitted clean version of the manuscript now read “Flow characterization and structural alterations in Ahmed glaucoma FP7 tubes after in-vitro aging in silicone oil”  3. The entire abstract was re-formatted and revised to clearly identify the aim of the study and its significance, communicate methods succinctly, and identify key results in a concise manner.  4. Lines 50-62 of the submitted clean version of the manuscript now read “Baerveldt glaucoma implant (BGI) and Ahmed Glaucoma valve (AGV) are the two most commonly used GDDs worldwide and use a silicone-based tube [2–4]. Although GDD tube shunts are traditionally inserted into the anterior chamber (AC), alternative locations, such as the ciliary sulcus or vitreous cavity, are being used more frequently for patients with anterior segment pathologies [5,6].  Posterior segment complications following GDD implantation have been frequently reported in the literature [7–11]. Of those, retinal detachment is reported in up to 5% of cases and carries significant visual morbidity [8,10,11]. Patients with previous ocular histories of uveitis, trauma, lattice degeneration, and vitreous syneresis may be predisposed to retinal detachment following GDD surgeries [10]. Silicone oil (SO) is often instilled intraocularly as a tamponading agent to treat retinal tears or detachments [12]. However, prolonged SO endotamponade may also result in SO emulsification, which can migrate beyond the posterior segment into the AC, thus altering the functionality of GDDs.[13]”  Lines 63-70 of the submitted clean version of the manuscript now read “Given the risk of concomitant SO and GDDs, it is important to evaluate their interaction. Previous studies report that eyes with SO endotamponade undergoing Ahmed glaucoma valve (AGV) implantation have a higher risk of surgical failure compared to eyes without SO [14–16]. However, the underlying reason for this phenomenon is unclear. The proposed hypotheses include chronic inflammation secondary to the presence of SO [14,15], migration of SO in the subconjunctival space through the tube [17], and lastly, formation of a diffusion barrier between the AC and the posterior chamber (PC). In contrast, few studies [7,10,18,19] and case reports [20–22] have described the successes of GDDs, including AGV, in eyes with SO [21].”  Lines 71-72 of the submitted clean version of the manuscript now read “Although clinical observations are helpful, they do not capture all the physical changes that may occur when two silicone-based products interact for a prolonged period [23]’  Lines 80-84 of the submitted clean version of the manuscript now read “As such, the gap our study aims to address is to characterize the dimensional changes in silicone-based FP7 AGV tubes and the resultant alterations in the flow dynamics of FP7 tubes once they are exposed to SO. The results of our study may offer potential explanations for the high failure rates of GDDs in eyes with SO instillation.”  5. Lines 96-98 of the submitted clean version of the manuscript now read “90.5 days of chronological aging at physiological temperature, corresponding to the accepted duration of SO endotamponade for retinal detachment (3 months) [12] [32].”  6. Lines 98-100 of the submitted clean version of the manuscript now read “To account for any stochastic effects due to accelerated aging, we also added a ‘heat control’ by aging tubes at 37°C for 13 days in BSS”  7. Lines 215-221 of the submitted clean version of the manuscript now read “Changes in luminal flow rates likely have a greater clinical significance in non-valved GDDs that lack any other means of proximal flow restriction. Nonetheless, while AGV has lower rates of post-operative hypotony due to a valve mechanism [2], AGV’s valve is also composed of silicone and may be subjected to interactions with SO. As such, migration of SO through the tube may affect the drainage of aqueous fluid by increasing the lumen size, as observed in our experiment, and by potentially changing valve function [54]. While beyond the scope of our current work, these complex interactions would benefit from additional future investigations.”  8. Lines 226-228 of the submitted clean version of the manuscript now read “Furthermore, the generalizability of our results is limited by the fact that we used silicone tubes only from the AGVs, which may differ in composition from silicone tubes in other GDDs.”  9. Please see the revised terminology throughout the manuscript and the figures.  10. Lines 135-146 of the submitted clean version of the manuscript now read “The mean percent change ± standard deviation (SD) in the length of tubes was 5.94 ± 2.0% and 5.55 ± 1.1% for media 1000cs and 5000cs SO, respectively. Tubes aged in BSS increased by 0.38 ± 1.6%, which was significantly different from the 1000cs and 5000cs SO tubes (P < 0.05 for both), but not the heat control tubes (P = 0.51), the latter of which slightly decreased in length by -0.42 ± 1.1%. The mean ± SD inner lumen area expanded for tubes in 1000cs and 5000cs SO by 11.75 ± 1.7% and 2.70 ± 0.6%, respectively, but contracted for tubes in BSS by -2.70 ± 2.7% (P < 0.01 for 1000cs SO vs. BSS; P = 0.068 for 5000cs SO vs. BSS). Similarly, the mean ± SD outer lumen area expanded by 10.80 ± 0.3% and 5.24 ± 5.4% for tubes in 1000cs and 5000cs SO, respectively. Whereas tubes in BSS and heat control tubes contracted by -7.87 ± 1.0% and -1.36 ± 1.7%, respectively. When compared to the BSS at 65°C group, the differences in mean changes of outer lumen area were statistically significant (P < 0.01 for 1000cs SO; P = 0.048 for 5000cs; P < 0.01 for heat control tubes) (Figure 3).”  Lines 155-158 of the submitted clean version of the manuscript now read “Post-aging at 65°C, the mean ± SD flow rates increased for 1000cs and 5000cs SO tubes by 61.0 ± 46.5% and 98.6 ± 58.9%, respectively. In contrast, flow rates decreased for BSS tubes by -4.92 ± 8.6%, which was statistically significantly lower when compared to 1000cs and 5000cs SO tubes (P < 0.01 for both)”  11. Lines 184-197 of the submitted clean version of the manuscript now read “S SO-induced expansion of AGV tube lumens is potentially clinically significant as it can impact the rate of aqueous fluid drainage and may facilitate SO migration. Our results suggest that 1000cs SO may pose an increased risk of sub-conjunctival SO migration due to its ability to permeate through the FP7 tube matrix readily. Previous studies have suggested that this migration may occur in various ways, including through the tube itself, through poorly sutured sclerotomies, or via leakage between the tube and the sclera [39–41]. Some studies have also reported a higher rate of in vivo emulsification of 1000cs SO oil than 5000cs [19,42,43], which may further enhance SO migration. Future studies may emphasize the role of SO viscosity in the success rates of AGV.  Moreover, our study indicates that non-silicone-based GDDs may be a better choice for refractory SO-induced glaucoma. One such example is EX-PRESS® (Alcon Inc., Fort Worth, TX, USA) metal shunt, which avoids SO-silicone rubber interactions altogether and clinically has shown similar or higher success rates compared to AGV [44–46]. Notably, because SO can cause Glaucoma through more than one mechanism [13], more clinical studies are needed to validate this option further.”  Lines 215-221 of the submitted clean version of the manuscript now read “Changes in luminal flow rates likely have a greater clinical significance in non-valved GDDs that lack any other means of proximal flow restriction. Nonetheless, while AGV has lower rates of post-operative hypotony due to a valve mechanism [2], AGV’s valve is also composed of silicone and may be subjected to interactions with SO. As such, migration of SO through the tube may affect the drainage of aqueous fluid by increasing the lumen size, as observed in our experiment, and by potentially changing valve function [54]. While beyond the scope of our current work, these complex interactions would benefit from additional future investigations.”  12. Lines 222-226 of the submitted clean version of the manuscript now read “In clinical practice, the drainage of aqueous flow through GDDs depends on several factors such as the location of the tube insertion, kinking of the tube [55], age of the patient, extent of fibrous encapsulation of the bleb [56], and protein content of the aqueous humor [2] – all of which can potentially confound a direct comparison of flow rates.”  13. Lines 230-236 of the submitted clean version of the manuscript now read “Overall, this controlled laboratory experiment showed that prolonged exposure of silicone-based AGV tubes to SO impacted the dimensions and flow characteristics of the tubes. Surprisingly, it did not impede flow through the FP7 tubes and, in fact, increased flow due to the increased diameter of the lumen. The increase in flow rates may carry clinical implications such as post-operative hypotony, especially for non-valved GDDs. Future studies may expand on our results by investigating the impact of SO exposure on different GDDs’ functionality using an *in vitro* model which may offer additional insights regarding the changes in flow dynamics.”  14. Please see the revised and restructured manuscript.  15. Please see our response to comment #9. |
|  |  |  |
